# 

**Published:** 1883-05

**Authors:** 


ILLINOIS STATE MEDICAL SOCIETY.

The Illinois State Medical Society will meet in Peoria, Ill., on the third Tuesday in May, 1883, at ten A. M. The reunion usually continues for three days. The following is a

LIST OF THE STANDING COMMITTEES.

*Practice of Medicine*:—N. S. Davis, of Chicago; B. M. Griffith, of Springfield: J. F. Todd, Chicago.

*Surgery*:—J. E. Owens, Chicago; J. T. Stewart, Peoria; M. Reece, Abington.

*Obstetrics*:—E. L. Herriot, Jacksonville; G. W. Jones, Danville; E. A. Ingersoll.

*Gynæcology*:—David Prince, Jacksonville; C. Chenowith, Decatur; E. S. Norred, Lincoln.

*Ophthalmology and Otology*:—S. J. Jones, Chicago; J. P. Johnson, Peoria; J. G. McKinney, Barry.

*Drugs and Medicines*:—T. J. Pitner, Jacksonville; Herbert Judd, Galesburg; P. H. Garriston, Macomb.

*Necrology*:—E. Ingals, Chicago; William Hill, Bloomington.

SPECIAL COMMITTEES.

*Simple Renal Catarrh*:—I. N. Danforth, Chicago; Washington West, Bellville.

*On the Practicability and Desirability of Separating the Work of Teaching in Medicine and Licensing to Practice*:—D. S. Booth, Sparta; E. P. Cook, Mendota; M. A. McClellan, Knoxville.

*The Diagnostic Peculiarities of Malignant Growths*:—Christian Fenger, Chicago.

*Analysis of a Certain Class of Remedies Concerning which Physicians are not Positive as to their Therapeutic Value*:—W. L. Ransom, Roscoe.

COMMITTEE OF ARRANGEMENTS.

J. Murphy, Chairman; A. S. Adams, J. H. Reeder, J. P. Johnson, I. T. Stewart, T. M. McIlvain, *ex officio*.

